# Research publications in the field of health: omission of hypotheses and presentation of common-sense conclusions

**DOI:** 10.1590/S1516-31802006000400011

**Published:** 2006-05-04

**Authors:** Egberto Ribeiro Turato, Alexandre Cason Machado, Douglas Fini Silva, Guilherme Machado de Carvalho, Natalia Reis Verderosi, Thiago Ferreira de Souza

**Keywords:** Medical education, Knowledge, Methods, Qualitative research, Research design, Educação médica, Conhecimento, Métodos, Pesquisa qualitativa, Projetos de pesquisa

## Abstract

**CONTEXT AND OBJECTIVE:**

Medical literature should consist of knowledge applicable to professional education; nevertheless, the profusion of articles in databases provokes disquiet among students. The authors considered the premise that scientific production in the field of health follows a mechanical description of phenomena without the clarity of motivating questions. The aim was to interpret material from expert reports, applied by medical students to analyze articles from renowned journals.

**DESIGN AND SETTING:**

This research project was exploratory, searching for latent meanings regarding methodological problems in a sample of papers. It was performed in a Brazilian medical school.

**METHODS:**

The sample was intentionally built, consisting of articles related to original research in the field of health, published over the previous five years. The results came from text content analysis, performed by a professor and his medical students.

**RESULTS:**

(1) Failure to state a hypothesis is an equivocal practice: articles did not show clarity of hypothesis to demonstrate that their authors had epistemological knowledge of the methods chosen. (2) There is a certain belief that in normal scientific practice, hypotheses are unnecessary: studies without explicit hypotheses led to suppositions that they merely repeat dominant models. (3) Presentation of common sense as scientific conclusions: research brings together what would have mobilized the researchers initially.

**CONCLUSIONS:**

Absence of formal hypotheses leaves scientific production vulnerable when put under epistemological discussion. Conclusions from scientific articles are often confounded with common-sense statements. Quantitative research is suggested, for studying the frequency of occur-rence of these dubious methodological points.

## Resumo

**CONTEXTO E OBJETIVO:**

A literatura médica deve se constituir de conhecimentos aplicáveis à educação profissional. Não obstante, a profusão de artigos nas bases de dados provoca inquietação entre os estudantes. Os autores consideraram a premissa de que a produção científica de campo em saúde segue uma descrição mecânica dos fenômenos sem clareza de questões motoras. O alvo foi interpretar material dos pareceres aplicados por estudantes de medicina ao analisarem artigos de periódicos indexados.

**DESENHO E LOCAL:**

Projeto de pesquisa exploratório, procurando significados latentes referentes a problemas metodológicos em uma amostra de artigos. Foi realizado em uma faculdade de medicina brasileira.

**MÉTODOS:**

Amostra construída intencionalmente com artigos de pesquisas originais de campo em saúde, publicados nos cinco anos precedentes. Resultados da investigação vieram de análise de conteúdo dos textos, realizada pelo professor com seus alunos.

**RESULTADOS:**

(1) *Omissão da enunciação de hipóteses é prática equivocada*: os artigos não mostraram clareza nas hipóteses para evidenciar que seus autores conheciam epistemologicamente os métodos escolhidos. (2) *Premissa de que a prática da ciência normal pode deixar de pensar hipóteses*: trabalhos sem hipóteses explícitas fizeram supor que eram meros repetidores dos modelos dominantes. (3) *Apresentações do senso comum como sendo conclusões científicas*: as pesquisas trouxeram fechamentos não-amarrados com o que teria mobilizado os investigadores inicialmente.

**CONCLUSÕES:**

Ausência de hipóteses formais faz produções científicas ficarem vulneráveis quando postas sob discussão epistemológica. Conclusões de artigos científicos são confundidas freqüentemente com afirmações do senso comum. Pesquisas quantitativas são sugeridas para estudar freqüências desses pontos metodológicos duvidosos.

## INTRODUCTION

Considerations about the quality of medical education have become more manifest in the scientific literature recently, resulting in many refined assessment methods for this purpose.^1^ To understand such processes better, students’ profiles must be considered. Factors involved may include: early choice of a medical career, persistence in taking school exams many times if necessary, awareness of difficulties and limitations in developing their careers, strong valuation of humanistic aspects of medicine, openness to new experiences, deep personal identification with the choice of profession, critical need for fulfillment in students’ careers, and conscious and unconscious desires to both help people and be recognized for their usefulness.^2^ From the outset of the medical course, pupils are exposed to many kinds of problems and they must be stimulated to search, under tutor supervision, for pertinent information in biomedical, clinical-epidemiological and psychosocial literature, in order to solve them. Likewise, there is the fact that the students face contemporary educational challenges in order to graduate as physicians with intellectual and ethical stature.

The present article originated from reflections on moments within the development of the diverse knowledge that has historically marked medical practice. It took into consideration the knowledge that has been transmitted in schools, to educate doctors, and reached contemporary issues within so-called scientific medicine. The present article thus brings the results from a learning-research activity performed among medical students at Universidade Estadual de Campinas.

Changes within the medical and scientific scenario have led to curricular modifications in many places. The authors, who were second-year students, and their professor were motivated to express their concerns about the profusion of scientific articles available from literature databases for consultation and learning. From a certain viewpoint, many of these articles present serious gaps with regard to relevant methodological reasoning. It is known that there are various pitfalls in both designing and conducting clinical research, and these include lack of randomization, lack of concealment, lack of blinding, and errors in hypothesis testing.^3^

Particularly, there are papers in which the research suffers from a lack of explicitness, starting from the initial phases of such investigation projects within the field of health. Overall, the research suffers with regard to its motivational questions, i.e. the working hypotheses that lead toward the search for answers. Such movements must necessarily occur for accurate academic research to emerge. The gaps mentioned earlier also relate to articles from projects that have been finished with the presentation of various conclusions that are far from having scientific rigor. These two uncertain points within scientific methodology raise an epistemological debate, from which the present writing came.

Beginning in 2001, with the implementation of a curricular reform within Universi-dade Estadual de Campinas, students began to follow a course module with the name “Introduction to Scientific Practice” (ISP). Currently, this takes up part of the first four semesters in the medical school. These modules were included in the medical teaching program with the aims of both educating health professionals with more precise notions about how scientific knowledge is produced and, particularly, making them critical judges/consumers of the abundant literature production available in large numbers of health journals. It would also make them critical judges of compendiums, presentations at medical congresses and classes at university.

Although the medical literature can be considered to be a tool for professional and personal education, it is not rare for the current medical and health information explosion to confound students and even teachers. With due regard to this, the curricular program of the fourth semester of the Unicamp medical course contains the ISP-4 module. Its suggestive sub-heading is “Notions of scientific history and epistemology applied to medical knowledge”. The construction and the writing of the present paper took place at the end of this module, as an accumulation of the respective reflections and experience.

The module begins with a summary of concepts from Greek science and Galileo, and continues up to the academic thought of today. It speaks about both cartesianism and positivism limiter actions in medical sciences. It presents the lineage from Galilean thought to the Bernardian principles that shape the current medical-epistemological construct. Prominence is given to the philosophical debates by Popper, Kuhn and Nietzsche. It also presents both external and internal factors relating to science, in the construction of medical knowledge. Epistemology is applied to the reading of articles in journals, medical handbooks and compendiums, and to the compilation of initial scientific drafts, dissertations and theses, and, finally, to the understanding of lessons in classrooms.

Likewise, the module mentions notions of the theoretical constructs in both anthropology and psychoanalysis, and their influence on the theory and practice of medicine. It notes experimental, epidemiological and humanistic research and their presence in the field of medicine, with a historical and critical viewpoint. Furthermore, it focuses on the locus of medicine within the academic field, with regard to the scenario of a *continuum* from the hard sciences to the humanities. It also does not neglect the problems relating to desubjectivation, dehistoricization, designification, desymbolization and sociological decontextualization in medical research. In the same way, it discusses the crucial role of interpretation by researchers in medicine. It concludes with a debate on both the strength of the *validity* and the frequent controversies about the results from the generalization of knowledge production in the field of health, i.e. the *reliability*.

## HYPOTHESES AND OBJECTIVE

The *premise* of this teaching-research activity work was formulated by the teacher of the module cited (in which scientific investigations were discussed with the medical students) and increasingly shared by the students during the course of their empirical contact with the literature that was put under examination.

The *initial and core hypothesis*, which was matured and formulated in relation to the material under examination, was that the current scientific production in the field of health research that is published in prestigious indexed journals is concerned with setting objectives consisting of merely mechanical descriptions of phenomena, with practically inertial continuity. It lacks clarity in the questions that mobilized the authors such that creative responses for building original knowledge might be given. The *derived hypothesis*, also formulated from the progress in the teaching-research activity, was that the absence of delimiting hypotheses for transforming the research into concrete form would lead to the presentation of scientifically feeble conclusions that would be more along the lines of common sense.

The *objective* of the present article, therefore, was to gain knowledge of and interpret some material from the scientific literature, which would be considered at home and in the classroom. The model to be utilized was that of a technical report, started by means of a checklist. This would be applied to recent articles selected intentionally from distinguished health journals. It was envisaged that the medical students would be exposed to several learning problems, and that these should encourage them to search the health literature critically.

The study was arbitrarily *delimited*, in its objectives, to Brazilian journals. The students made assessments of these journals as part of their curricular activities, followed by systemized writing (about the cited expert's report). This consisted of multiple evaluative items. These students were faced with the task of detecting the explicit presence of working hypotheses in the published research works. Any such hypotheses were highlighted at that time. Also, the papers were examined with regard to the character of any pertinent conclusions with which these publications culminated, and these were also highlighted.

## METHODS

It was sought to address the abovementioned curricular requirements critically. Thus, this module was put into operation as a series of themes, along the lines of the course program themes outlined in the Introduction. The strategy utilized involved theoretical lessons given in alternation with presentations, which were oral and written presentations concomitantly. The latter were produced by the students, in relation to methodological and epistemological evaluation of the scientific texts, which were chosen on that occasion from among the articles in renowned academic journals.

The assessments were carried out by 24 teams, with five students in each team, week by week. Two teams (ten students) evaluated each article, using the model of the expert's detailed report that had been adapted by their teacher for this purpose. These articles consisted of 35 assessment items in all, and these were distributed into eight parts that were to be assessed: title of the study, abstract and key words; section containing introductory questions; section with hypotheses and objectives; section with subjects and methodological resources; section with results and discussion; section with conclusions, recommendations and suggestions; bibliographical references; and content balance and report style.

Taking advantage of the intrinsic nature of this educational activity, the project of scientifically preparing the available material unfolded naturally. The methodological design of the teaching-research activity was qualitative,^4^ i.e. it was marked by a search for *latent meanings in the findings*.^5^ This research took place as an in-depth contact with the texts selected for study. Random sampling, as found in quantification studies concerned with studying the frequencies of features present in texts or causal correlations between verified situations, elements, etc. was not pertinent.

In this strategy, the results were the fruit of the *content analysis technique* applied to the set of scientific articles. This technique was introduced during the second semester of 2004, thus time-limiting the data collection. In the analysis, the *constructed categories* for discussion were methodologically started just after using the *theoretical data saturation technique*: “*the point in category development at which no new properties, dimensions, or relationships emerge during analysis*”.^6^ It was assumed that new and successive data collections (from assessing articles in the classroom and outside) were not bringing in substantially different elements for arriving at plausible theories, i.e. for understanding the underlying logical order.

Following recognized principles, in contrast to what would occur in quantitative studies, the publications analyzed were not taken probabilistically, since the aim was not to gene ralize mathematical results. Three particular features were intentionally put forward: these were the *inclusion criteria*, which referred to the objective of the delimitation that was being studied. These criteria were that the texts should be: (a) publications extracted from original research; (b) designed as health investigations; and (c) from the last five-year period. In this approach, the feasible *generalization* is the one relating to conclusive *concepts*, which can then be utilized by readers/consumers of the research report in their future situations of contact with other scientific productions.^7^

It was during the presentation of the systematized reports, to the whole group, within a discussion of the methodological limitations perceived in the articles, that two *topics* caught the students’ attention. There was repeated omission of any mention of hypotheses, and many conclusions only had weak logical pertinence to the work produced. Because this sample had already been constituted, another twelve publications gathered from indexed journals in the Brazilian database Scientific Electronic Library Online (SciELO)^8^ were therefore also analyzed. These were *papers* taken from original research conducted within the fields of both clinical and psychosocial investigations applied to health.

It is well known that the *choice of a research objective* is a strongly scientific-political decision, and not a predominantly scientific-methodological one, as many people naively think. Therefore, it would be appropriate to promote an ideological debate about why a database of exclusively national scope was chosen, but not why a bibliographical field mixed with any database of worldwide range was the one selected by the authors. In this first study, the researchers arbitrarily decided to consider their own country's relevant points of bibliographical production.

## RESULTS

The main result from the present study was that the researchers found texts that, despite coming from original research, did not include clear *working hypotheses*. That is, there was insufficient clarity to demonstrate to appraisers and readers that, from an epistemological point of view, the authors knew what the correct methodological arrangement among the diverse and possible ones for health sciences would have been. The lack of *mention of such hypotheses*, which would have guided their authors toward the scientific enterprise, caused concern among the students during the evaluations in the classroom.

On the other hand, it seems that there is a certain premise that it is normal scientific practice, within the same paradigm, *not to think about hypotheses*. This emerged from earlier discussions, and related to our first presupposition. Investigative studies without explicit hypotheses give rise to the supposition that these enterprises have a merely mechanical course. That is, they uncritically repeat the dominant group's methodological models in the world of academic medicine.

Amongst the results found, a third relevant point that was criticized by the student investigators was that many conclusions were unconnected to the working presupposition. They neither agreed nor reviewed them, but simply did not present them. It was observed that, without a continuing strand of meaning, the articles went towards their conclusions without any ties to the ideas that had mobilized the researcher initially. This gave rise to “conclusions” that seemed more to emerge from authors’ feelings and to be shaped by *common sense* introduced by the sociocultural environment. In other cases, the final considerations even arrived at *moral conclusions.*

## DISCUSSION

### 1. Failure to state a hypothesis is an erroneous practice

The term “hypothesis” was understood in this text as coming from the Greek ηψπηοτεσισ (*hypothesis*), signifying “basis, supposition”. It is composed by ηιπο (*hypo*), “under”, and τιτηεναψ (*tithénay*), “to put or set down”, which literally means the thing that is taken as the basis. In a broad sense, it brings the idea of something placed merely provisionally in order to guide investigations.^9^ Theoretically, the names given to this concept would matter little: problem, question, hypothesis, presupposition, premise, conjecture, postulate, guess, suspicion, suggestion, anticipation, and so on.

Therefore, directly addressing the presentation of the research objectives, although the texts alleged that the investigative presuppositions would be implicit, they did not ensure clarity at a scientific level. Failure to present hypotheses, before enumerating the objectives, usually represented a failure to respect the *logical sequence of stages*, which are understood as occurring naturally in the mind of the thinker. The texts studied gave the idea that the authors had established the targets to be reached in their scientific plans, but the articles did not show that those authors had in fact started from premises that were of concern to them.

Many people take the understanding that a hypothesis is born from observed data. This, however, is a belief that such hypotheses are in consonance with a positivist conception. Modern epistemology supports the idea that, in fact, hypotheses are imagined as phenomena, or even *invented by the human mind*. At this point, to search previously for postulates is not the same as making unnecessary digressions. It is, however, a way of using them as an indispensable tool for understanding such erroneous practices that are put into how scientific research is conducted.

To deepen this discussion, Comte should be recalled. This writer ambiguously defended the *positivist doctrine* that the thought should be guided by the rigor of two fundamental rules (1844): *any proposition which is not ultimately reducible to the simple enunciation of a fact, whether particular or general, does not present any real intelligible sense*. Moreover, *pure imagination must lose its mental supremacy, and be subordinate to observation*.^10^ In this light, scientists should take the philosopher Nietzsche into consideration. In perhaps the most incisive critique of the positivist conception, he deconstructed it thus (1886): “*Against the positivism that halts at phenomena — ‘There are only facts’ — I would say: no, facts are just what there aren't, there are only interpretations. We cannot determine any fact ‘in itself’: perhaps it's nonsensical to want to do such a thing*”.^11^

To put this debate even more into context, it is pertinent to remember that, historically, the Greek conception of science was contemplative and did not hold with experiment. Modern science, whose father is Galileo, came to into being when this thinker characterized scientific knowledge as the knowledge that is found in codified form in nature (1623): “*Philosophy is written in this grand book, the universe, which stands continually open to our gaze. But the book cannot be understood unless one first learns to comprehend the language and read the letters in which it is composed. It is written in the language of mathematics, and its characters are triangles, circles, and other geometric figures without which it is humanly impossible to understand a single word of it; without these, one wanders about in a dark labyrinth*”.^12^

By breaking the link between knowledge and religion, which had been a source of structured knowledge about nature, and by breaking with the Aristotelian philosophy that had been used by European university professors until then, Galileo, with Copernicus, Kepler and, later, Newton, created the biggest revolution within Western thinking and, hence, they gave autonomy to science. Einstein praised the Italian thinker in this manner: “*The discovery and use of scientific reasoning by Galileo was one of the most important achievements in the history of human thought.* […] *Galileo's contribution was to destroy the intuitive view and replace it by a new one.* […] *The transition from Aristotle's line of thought to that of Galileo formed a most important cornerstone in the foundation of science*”. Also, according to Einstein, the founder of science again attested to his genius, when he formulated the problem of determining the speed of light, even though he could not solve it, since he had little chance of doing so with the experimental techniques available at that time.

The winner of the 1912 Nobel Prize for Physics brilliantly laid out his thoughts about the locus of a hypothesis, the matter that is under discussion here: “*The formulation of a problem is often more essential than its solution, which may be merely a matter of mathematical or experimental skill. To raise new questions, new possibilities, to regard old problems from a new angle, requires creative imagination and marks real advance in science.* […] *The importance of seeing known facts in a new light will be stressed and new theories described*”.^13^

From these great scientists’ contributions, a good scientific publication today should ideally make the reader glimpse different relationships. This fascinates, because such relationships can be understood in the way that led Newton to state that “*If I have seen further it is by standing on the shoulders of Giants*”, in a letter to the physicist Robert Hooke in 1676.^14^ If research of today, including medical investigations, makes us watch over a greater distance, this occurs because its authors stand on the shoulders of revolutionary thinkers, such as Galileo, in order to develop their themes in greater depth.

From knowledge of other types of work, although outside of the scope of the present work, it is known that bench/laboratory research is perhaps the only conspicuous exception to the infallible mention of hypotheses. However, in these studies, if hypotheses were invariably present, at the other extreme, they would often arrive on the scene as an automatic ritual. To cogitate whether the phase of putting forward hypotheses would have settled into a routine of operational rules (and why), in the case of experimental series currently conducted at famous university centers, would fit within epistemological investigations.

### 2. A certain premise that normal scientific practice fails to think of hypotheses

It has already been seen that researchers are setting out their objectives in health-related studies, without having stated what the premises were that established the purpose of their work. This led the present authors to formulate a *working hypothesis* regarding why *these researchers’ hypotheses* did not appear in either their project designs or their final reports. The physicist and famous historian of science, Kuhn, should be cited here. According to Kuhn's acclaimed doctrine, *normal science* is work carried out in accordance with the academic community's norms, within a certain paradigm, while *revolutionary science* is the type that breaks with the scientific paradigm in force, thereby destroying the linearity of a certain domain of accumulated knowledge.^15^

It is very important to underline Kuhn's delimitation of what paradigms in science are (1962): *universally recognized scientific achievements that for a time provide models for problems and solutions to a community of practitioners*.^15^ In this way, authors of works within a major paradigm (an umbrella for academic production) find not only themes for their research, but also ready solutions. There only remains the patient physical and intellectual effort required for solving the puzzle of scientific work, with conclusions that are far from unforeseeable. In the game of scientific production practiced by institutions, the announcement of hypotheses may, lamentably, have become superfluous, because it is known beforehand where the scientific enterprise will arrive, without having to think hard about it.

Finally, it is crucial to emphasize that *there is no thesis without hypothesis*. There is no scientific work without intelligent and moving questions, whether or not these are clear within the researcher's consciousness, and whether or not these are an express part of the research project or the writing up of the final report.^16^ Since all thoughts begin with a problem, it is legitimate to take the understanding that health researchers who are unable to either perceive or formulate such problems are not prepared to bring science into this field. In our culture, the teaching processes in the medical sciences are, unfortunately and commonly, more often concentrated on training students to respond through both classified diagnoses and standardized treatments. However, the teaching of sciences in any area of human knowledge fails in a situation in which solutions are given for questions that were neither formulated, nor even understood.

Traditional methodologists have not avoided characterizing science as being grounded on a set of principles. In these, they show that *hypotheses* serve for developing predictions, including in the medical sciences, in order to submit them to experimental tests or observation.^17^ It has become conventional wisdom that experimental research (and quantitative studies in general) follows deductive reasoning, whereas field research (and qualitative studies in general) follow inductive reasoning. From an epistemological perspective, this division is artificial, since in fact the elaboration of knowledge in science occurs within an inductive-deductive process spiral. Nonetheless, the extent to which hypotheses are the capital for human movements for producing scientific knowledge can be seen from Table 1.

**Table 1.** The movements of a researcher during the course of a scientific enterprise

*Provokers of questions:* empirical reality, scientific literature	Generalization of concepts (humanistic research, qualitative research)	← New settings, new situations
↓		↑
Hypotheses formulated		Hypotheses reviewed
↓		↑
Incipient theory		Theory elaborated
↓		↑
Field observed		Analysis of results
↓		↑
Deductive reasoning	→ Data collected →	Inductive reasoning
	↓	
	Hypotheses tested	
	↓	
	Generalization of mathematical results (experimental research, quantitative resea rch)	

### 3. Inappropriate presentations of common sense as scientific conclusions

Failure to present scientific conclusions in the true sense of the word demonstrates that the authors did not create a rupture between common sense and the knowledge built up through the scientific activity. The leap from naivety to scientific attitudes indicates that there has in fact been both understanding and adoption of the scientific code. This contributes to the idea that researchers did not go out from the their immersion in popular reasoning to head towards the *coded language of science*. This interpretation gains plausibility from phrases that were encountered in the articles examined, which gave rise to ideas similar to the following ones:

- *“New studies must be performed in relation to this subject in order to have deeper and greater understanding of the problem.”*
- *“More efficient ways for therapeutic follow-up of patients must be investigated.”*
- *“The discussion of the present study needs to be evaluated in relation to both the reach and limits of the method used.”*
- *“Relationships in health services need to be humanized.”*
- *“There is a need for health service users to be managed by a multiprofessional team.”*
- *“It was evident that a support network for people who experience health problems is important.”*
- *“Many aspects of this disorder could be prevented through health promotion actions.”*
- *“Persons affected by this syndrome also have the right to experience rewarding sexuality in the way that normal people do.”*
- *“Campaigns against tobacco are indispensable in university environments, especially among health personnel.”*
- *“It becomes necessary to make the population aware about this kind of health problem.”*

In a critical discussion, it has to be asked whether the opposite of these statements would be defensible. Would they be affirmations that actually uncover an invisible order, in the way that science demands? Would they have been thought of from a non-naive viewpoint? Do they really consist of explanatory or comprehensive theories on phenomena? The question is how to avoid the pitfalls of a certain arrogant childishness, through creating a hierarchy of levels of knowledge by utility, in which scientific knowledge would be placed above others such as common sense. Overall, the question also emphasizes that it is not sensible to spend human energy, financial resources and precious time on conducting research that comes to conclusions that both were already constructed empirically and are in the lay domain.

Popper, one of the greatest epistemologists of the twentieth century, stated that scientific facts are those that *can be contradicted*. The *truth*, as the conclusion of a research enterprise, is something that must be left open. This is not because there is always other knowledge to be written up and thereby added to the preceding knowledge (in the manner of bricks laid in the wall of science). Rather, it is open because there will always be a possibility of finding cases or occurrences that *negate* a given existing theory and that these must therefore be used to review the theory. Popper defended the criterion of demarcation of *falsifiability* instead of *verifiability*: “*I shall not require of a scientific system that it shall be capable of being singled out, once and for all, in a positive sense; but I shall require that its logical form shall be such that it can be singled out, by means of empirical tests, in a negative sense: it must be possible for an empirical scientific system to be refuted by experience*”.^18^

In other words, only a search for *negative data* would lead to both reformulation of the initial theory and advancing of scientific knowledge, and not the search for *positive data*, which would only serve to confirm previous hypotheses. It suffices to examine the numerous scientific articles in databases to demonstrate, through a simple reading, that the rule has been “to find conclusions”. This only seems (when it occurs…) to confirm premises that had guided the scientific investigation (and how rare it is to see a doctoral thesis that concludes by refuting its own hypothesis!).

### 4. Addendum: students versus academic institutions’ contradictions

Contrary to what might be imagined, medical students have a certain independence with regard to perceiving both the *incongruence and incompleteness of scientific notions* that are presented to them. This is because they are novices regarding their acquaintance with the environment of scientific production. On the other hand, teachers and senior researchers may have a debilitated capacity to debate this, considering that they have long experience of both constructing and presenting knowledge. In the great academic world, because students are at a young age, they easily latch onto the *contradictions of human institutions*, in particular among scientific leaderships.

Those who work in the field of education know how to quickly bring out majestic professorial speeches that, not infrequently, are used to conceal underlying fragility. At first, official voices may proclaim, for example, their belief that reflection on the concept of medical education, in parallel with the evolution of medicine, is extremely profitable and elucidative. But at the next moment, with regard to a proposed related matter, these voices will be able to allege laconically and in a simple bureaucratic tone, that the proposition was not selected for the agenda because of the great number of other proposals that would have gained greater priority.

It therefore is up to the generations coming into universities to act as questioners, both to deconstruct the rhetoric of older generations and to dislodge them from repetitive academic production. Above all, it falls to the university as a whole, to fulfill its historical role of both *being a thinking entity itself* and *making society be equally thoughtful*. Universities must, within their ideals, have the noble function of teaching how to debate above the level of pragmatic function, while equally legitimately supplying professionals to the job market.

## CONCLUSIONS AND RECOMMENDATION

Failure to question is a deceitful habit both in drawing up research projects and in the methodological process. This leads to investigative enterprises becoming marked by a fragile basis in the epistemological debate. The practice of *automatic science* can lead to unreflective renunciation of working hypotheses that have really been thought through. It needs to be debated whether stimulating the illusory behavior of adding new facts to theories that are already present in the literature does not become harmful to good scientific impulses.

The current and celebrated profusion of publications within the health environment has an ambiguous nature. It brings in a fallacious vainglorious characteristic that is embedded in and often displayed by university leaders and academic centers of excellence. Without hypothesis there is no thesis, because no conclusions can be extracted from situations that did not have underlying ideas requiring confirmation, refutation or revision. Hypotheses are necessary for scientific tasks: to omit them is to leave the project imperfect.

In the articles examined, the usual situation was that the final propositions of the scientific work were marked by common sense. Thus, these articles diverge from their primordial vocation of uncovering the relationships underlying the manifest content. They are also trapped by the pitfall of repetition of opinions inherited from social tradition. It is therefore suggested that publishing committees of biomedical journals revise their expert reports in order to allow referees to make better evaluations of matters that are the focus of such articles.

Consequently, for those who have a duty towards or an interest in research, it is recommended that they initially try to plan at least two types of research: qualitative investigations performed using indexed publications of worldwide reach, such as Medline, and quantitative research in order to provide evidence regarding the frequencies with which phenomena relating to these methodological questions (that are incorrectly placed or unduly not placed in scientific articles) would occur.
